# High dietary Fructose Drives Metabolic Dysfunction-Associated Steatotic Liver Disease via Activating ubiquitin-specific peptidase 2/11β-hydroxysteroid dehydrogenase type 1 Pathway in Mice

**DOI:** 10.7150/ijbs.97309

**Published:** 2024-06-17

**Authors:** Chunlin Li, Meng Li, Wei Sheng, Wenjun Zhou, Ziqi Zhang, Guang Ji, Li Zhang

**Affiliations:** 1 Institute of Digestive Diseases, Longhua Hospital, Shanghai University of Traditional Chinese Medicine, Shanghai 200032, China.; 2 State Key Laboratory of Integration and Innovation of Classical Formula and Modern Chinese Medicine, China.

**Keywords:** dietary fructose, MASLD, hepatic steatosis, inflammation, USP2

## Abstract

Metabolic dysfunction-associated steatotic liver disease (MASLD) is the most common cause of chronic liver-related morbidity and mortality. Though high fructose intake is acknowledged as a metabolic hazard, its role in the etiology of MASLD requires further clarification. Here, we demonstrated that high dietary fructose drives MASLD development and promotes MASLD progression in mice, and identified *Usp2* as a fructose-responsive gene in the liver. Elevated USP2 levels were detected in the hepatocytes of MASLD mice; a similar increase was observed following fructose exposure in primary hepatocytes and mouse AML12 cells. Notably, hepatocytes overexpressing USP2 presented with exaggerated lipid accumulation and metabolic inflammation when exposed to fructose. Conversely, USP2 knockdown mitigated these fructose-induced changes. Furthermore, USP2 was found to activate the C/EBPα/11β-HSD1 signaling, which further impacted the equilibrium of cortisol and cortisone in the circulation of mice. Collectively, our findings revealed the role of dietary fructose in MASLD pathogenesis and identified the USP2-mediated C/EBPα/ 11β-HSD1 signaling as a potential target for the management of MASLD.

## Introduction

Metabolic dysfunction-associated steatotic liver disease (MASLD), which is the replacement term for non-alcoholic fatty liver disease (NAFLD), represents a predominant chronic hepatic disorder and affects an estimated two-fifths of the world population^1^, ^2^. The trajectory of MASLD spans from benign steatosis to a more aggressive form, metabolic dysfunction-associated steatohepatitis (MASH), and may progress to cirrhosis and hepatocellular carcinoma (HCC)^3^. Recently, the FDA approved Resmetirom as a pharmacologic choice for non-cirrhotic MASH patients with fibrosis at moderate to advanced stages. Still, lifestyle modification remains the principal therapeutic strategy for MASLD. Nutritional composition, particularly the interplay of carbohydrates, lipids, and proteins, is fundamentally linked to hepatometabolic integrity^4^. Notably, high fructose intake is increasingly recognized as a major dietary contributor to MASLD. The link between excessive fructose consumption, enhanced hepatic fat deposition, and insulin resistance (IR) has been well-established^5^, ^6^. A notable cohort study reported that high fructose intake elevated the risk of MASLD threefold, raising concerns about its significant impact on disease pathogenesis^7^. Conversely, strategic inhibition of fructose metabolism has shown therapeutic potential, as seen in a phase 2a clinical trial where PF-0683591 improved liver steatosis and inflammation in affected individuals^8^. Moreover, fructose permeates adipose tissue metabolism, as evidenced by altered insulin sensitivity, increased high-sensitivity C-reactive protein levels, and augmented lipid oxidation, potentially aggravating MASLD-related complications^9^. These findings advocate for a holistic approach in addressing fructose-related metabolic disruptions.

Within the alimentary tract, dietary fructose is swiftly assimilated through intestinal uptake and directed towards hepatic metabolism^10^. Unlike glucose, which is readily converted into glycogen for storage in the liver, fructose preferentially fuels *de novo* lipogenesis (DNL), culminating in lipid deposition in hepatic cells^11^. Excessive fructose exposure could also inhibit fatty acid oxidation in the liver, intensify IR, and elevate serum uric acid (UA) concentrations^12^-^15^. Nevertheless, the exact mechanisms by which fructose incites MASLD remain to be fully elucidated.

Recent investigations have highlighted the complex role of ubiquitin-specific peptidase 2 (USP2) in liver metabolic homeostasis. USP2 modulates glucose and lipid metabolism, insulin sensitivity, and inflammatory processes. Enhanced USP2 expression boosts glucose production and aggravates glucose intolerance in obese mice^16^. In macrophages, USP2 promotes inflammation by activating the nuclear factor kappa B (NF-κB) pathway^17^, ^18^. Moreover, USP2 encourages lipid absorption by enhancing low-density lipoprotein receptor (LDLR) stability and compromising the actions of an inducible degrader of the LDLR (IDOL)^19^. Importantly, by deubiquitinating CCAAT/enhancer binding protein alpha (C/EBPα), USP2 also regulates 11beta-hydroxysteroid dehydrogenase type 1 (11β-HSD1), thereby playing a vital role in the homeostasis of glucocorticoids within liver cells^16^.

In this study, we found that high dietary fructose consumption promotes MASLD development and progression in C57BL/6J mice, identified *Usp2* as a specific fructose-responsive gene, and revealed that the USP2-mediated C/EBPα/11β-HSD1 signaling is involved in disrupting cortisol homeostasis. Our results suggested that fructose-induced hepatic and metabolic disorders are intricately linked to the regulation of glucocorticoid homeostasis via the USP2/ 11β-HSD1 pathway.

## Materials and Methods

### Animals

Male C57BL/6J mice, 7 weeks of age, were purchased from GemPharmatech Co., Ltd (SCXK2023-0009). The mice were maintained in a specific pathogen-free environment, with a controlled temperature of 22 ± 1°C, humidity at 55 ± 15%, and a 12-hour light/dark cycle. All procedures were approved by the Animal Care and Use Committee of Shanghai University of Traditional Chinese medicine **(**PZSHUTCM2305210005**)** and complied with the established guidelines for the care and use of laboratory animals. Following a one-week acclimation period, the mice were randomly assigned into the following groups: Con, Fr, WD, WFr, and WDF groups, n=6/group. Con and Fr groups were fed with the chow diet (CD) (Harlan, TD. 08485); WD, WFr, and WDF groups were fed with the Western diet (WD) (Harlan, TD. 88137). Throughout the experiment, the Con and WD groups had access to tap water; meanwhile, the Fr and WDF groups were supplied with water containing high fructose corn syrup (HFCS)^20^-^22^, consisting of 23.1g/L d-fructose (Sigma Aldrich, G8270) and 18.9g/L d-glucose (Sigma Aldrich, F0127); and the WFr group was given tap water for the initial 10 weeks and HFCS-containing water for the subsequent 10 weeks. Food and water were provided ad libitum over the 20 weeks, and the average consumption of water and food per cage of mice was weighed and recorded every week. After 20 weeks, venous blood was collected for immediate supernatant extraction for subsequent use, livers were harvested and weighed, then either fixed promptly or snap-frozen and stored at -80°C.

### Isolation of primary mouse hepatocytes

C57BL/6J mice, 8-12 weeks old, were anesthetized with 2% pentobarbital via intraperitoneal injection. Thoroughly perfuse the liver through the inferior vena cava with EGTA buffer (136.89mM NaCl, 5.37mM KCl, 0.64mM NaH2PO4.H2O, 0.85mM Na2HPO4, 9.99mM HEPES, 4.17mM NaHCO3, 0.5mM EGTA, and 5mM glucose), enzyme buffer (136.89mM NaCl, 5.37mM KCl, 0.64mM NaH2PO4.H2O, 0.85mM Na2HPO4, 9.99mM HEPES, 4.17mM NaHCO3, and 3.81mM CaCl2.2H2O) containing 100CDU/mL collagenase (Sigma C0130-1G) sequentially, at a rate of 5mL/min for 3min and 7min, respectively. Tear up liver tissue and shake it thoroughly at 38°C for 15min, and filter it using a 100μM strainer. Then, repeatedly centrifuged liver tissue suspension at 50G for 2min to obtain fully dispersed hepatocyte particles. Plant these hepatocytes on the 6-well plates and routine cultivation with DMEM/F12(1:1) (Gibco C11330500BT) containing 10% fetal bovine serum (FBS) (Lonsera S711-001S), 10^-8^ M dexamethasone (Sigma d1756), 10^-8^ insulin (Macklin R917753), and 1% penicillin-streptomycin (Gibco 15140-122).

### Cell culture

Mouse primary hepatocytes and murine cell line AML12 cells (Shanghai Cell Bank, China) were cultured in DMEM/F-12(1:1) (Gibco C11330500BT) medium with 10% fetal bovine (Lonsera S711-001S) and 1% Pen Strep (Gibco 15140-122) at 37℃ in a Forma™ II WATER JACKET incubator (Thermo Scientific, USA) with 5% CO_2_. The cells were treated with FFA (PA 100μM: OA 200μM), Fr (fructose 88.8mM), or Cortisol (Sigma-Aldrich, St. Louis, MO, USA, 1μM), respectively.

### Transient transfection

Overexpression (OE) and knockdown (KD) of *Usp2* in cells were established with a plasmid (Genomeditech, China) (Table 1) via transient transfection. Transfection begins when the cell fusion rate is about 70%. Transfection solution is prepared according to the following steps: Firstly, the lipofectamine mixture, consisting of 20μL Opti-MEM (Gibco 31985-070) and 8μL Lipofectamine (Invitrogen 11668-019), was incubated for 5min. Next, the plasmid mixture, composed of 200μL Opti-MEM and 3.2ug plasmid, was prepared. Mix the above two for 20 minutes and add 400μL to each well. After 24 hours, the transfection solution was replaced with a regular culture medium for another 48 hours. The cells and their culture supernatant were harvested for the following detection.

### Biochemical indicators

The serum biochemical indicators, including triglyceride (TG), total cholesterol (CHOL), low-density lipoprotein-cholesterol (LDL-c), aspartate aminotransferase (AST), alanine aminotransferase (ALT), and alkaline phosphatase (ALP) were detected by a TBA-40FR automatic biochemical analyzer (Toshiba, Japan) according to the testing procedures of manufacturers. Commercially Mlbio ELISA kits were used to measure the levels of cortisol (#m1001959-2), cortisone (#m1460921), interleukin (IL)-6 (#m1063159-2), IL-1β (#m1301814-2), and tumor necrosis factor (TNF)-α (#m1002095-2); and the TG assay kit (Nanjing Jiancheng Bioengineering Institute #A110-1-1) was purchased to test the cellular lipid content.

### Histological examination

The histological examination was performed according to the established protocol^23^. **Tissue samples**: After conventional paraffin embedding, the tissue sections were cut evenly to 5μM thickness for H&E staining (Biosharp BL735B-2), and hematoxylin (Kohypath KH-HEMH-OT-500) was used to stain the nucleus. The middle part of the largest lobe of fresh liver were harvested and cut to 8μM thickness for oil red O (ORO) staining (Sigma Aldrich 00625), and hematoxylin was used to stain the nucleus. Paraffin-embedded blocks were cut into 3μM thickness for immunohistochemical (IHC) experiment: SABC immunohistochemical staining kit (Boster SA2002) was used, primary antibodies include F4/80 (CST 70076S, 1:300), CD68 (CST 97778, 1:300), and USP2 (Proteintech 10392-1-AP, 1:200). Photos were taken using Stratafaxs Ⅱ microscopes and cell imaging system (Tissuegnostics). **Cell samples**: Fresh cells were used for ORO staining^24^, and photos were taken under an AXIO Vert.A1 microscope (Zeiss) immediately. Fresh cells were used for Lipi-Red staining (Dojindo LD03), DAPI (Beyotime C1006) was used to stain the nuclear, and the operation manual was strictly followed. Immunofluorescence assay was performed as reported^25^. The primary antibody USP2 (Protein 10392-1-AP, 1:200) and fluorescent secondary antibody (Invitrogen A11304, 1:1000) were used. All these photos were taken under the Image X press^®^ Micro4 equipment (Molecular).

### Western blot

The protein sample was prepared^26^ and quantified using a BCA kit (Beyotime P0012). Express Plus™ PAGE Gels 4-20% (Genscript M42015C) was used for protein electrophoresis. Western blot experiment was performed on a Mini-Protean^®^ Tetra System electrophoresis apparatus (Bio-Rad, China) (60V) and a rapid film transfer instrument Electrophoresis (Genscript, China) (regular 10min), sequentially. After 30min of isolation in protein-free rapid blocking buffer (Epizyme, China), incubate the primary antibody (USP2, Protein 10392-1-AP, 1:500; C/EBP α, Abclonal A0904, 1:500; 11β-HSD1, Abclonal A1619, 1:500) overnight at 4℃. Incubation with the Goat anti-rabbit IgG (CST 7074s, 1:2000) at room temperature for 1 hour. Using an Omni-ECL™ Femto Light Chemiluminescence Kit (Epizyme, China), immunoblotting was visualized on the Tanon-5200 Chemiluminescent Imaging System (Tanon Science and technology).

### Real-Time Quantitative PCR (RT-qPCR)

The total mRNA was extracted using the Trizol (Invitrogen, Carlsbad, CA, USA) method. In short, RNA after concentration test on Nanodrop 2000 spectrophotometer (Thermo Scientific) was reversely transcribed into cDNA using the reverse transcription kit (Accurate Biology, China). qPCR was performed on Quantstudio^⑤^ Real-Time PCR System (Thermo Fisher Scientific) using the method of SYBR Green (Accurate Biology AG11701). Apply the 2^-ΔΔT^ method, the expression of target genes was relative to *β-actin*. Relative sequences of the primers (Shanghai Shanjin Biotechnology, China) were shown in Table 2.

### RNA Sequence (RNA-Seq)

Fresh liver samples (n=5/group) were collected for RNA-Seq^27^. RNA quality and concentration assessment were performed using a Nanodrop 2000 spectrophotometer (Thermo Fisher Scientific) and Agilent 2100 Bioanalyzer with a 2100 RNA nano 6000 assay kit (Agilent Technologies, USA), respectively. Post-quality control, eukaryotic mRNA with poly-A tails was enriched using the TIANSeq mRNA Capture Kit (Tiangen, China). Subsequently, transcriptome sequencing libraries were constructed from the enriched RNA using the TIANSeq Fast RNA Library Kit (Illumina, USA). Library quantification was conducted with a Qubit 2.0 fluorometer (Life Technologies) and diluted to 1ng/µL, followed by insert size validation on Agilent 2100 and precise quantification through quantitative PCR (Q-PCR) (library activity > 2nM). The index-coded samples underwent clustering with a cBot Cluster Generation System using a TruSeq PE Cluster Kit v3-cBot-HS (Illumina, USA), as per manufacturer protocols. Sequencing was performed on an Illumina platform, yielding 150 bp paired-end reads.

Data analysis was conducted on high-quality reads, filtered through internal Perl scripts. Gene expression levels were estimated using FPKM. DESeq2 R package was employed for differential expression analysis between groups, adjusting *p*-values with Benjamini and Hochberg's method to manage the false discovery rate. Genes were deemed differentially expressed with *p*<0.05 as per DESeq2. An adjusted *p*-value of 0.05 was the criteria for significant differential expression. The Cluster Profiler R package facilitated statistical enrichment analysis of differentially expressed genes within KEGG pathways.

### Statistical analysis

SPSS software (version 26.0; IBM, Armonk, NY, USA) was used to analyze all the data, the data were displayed as means *±* SD. Differential analysis between two or more groups were performed by Student's *t*-test and one-way analysis of variance (ANOVA), respectively. Statistically significance was set as *p*<0.05.

## Results

### High dietary fructose drives the development of MASLD

**Figure 1:**
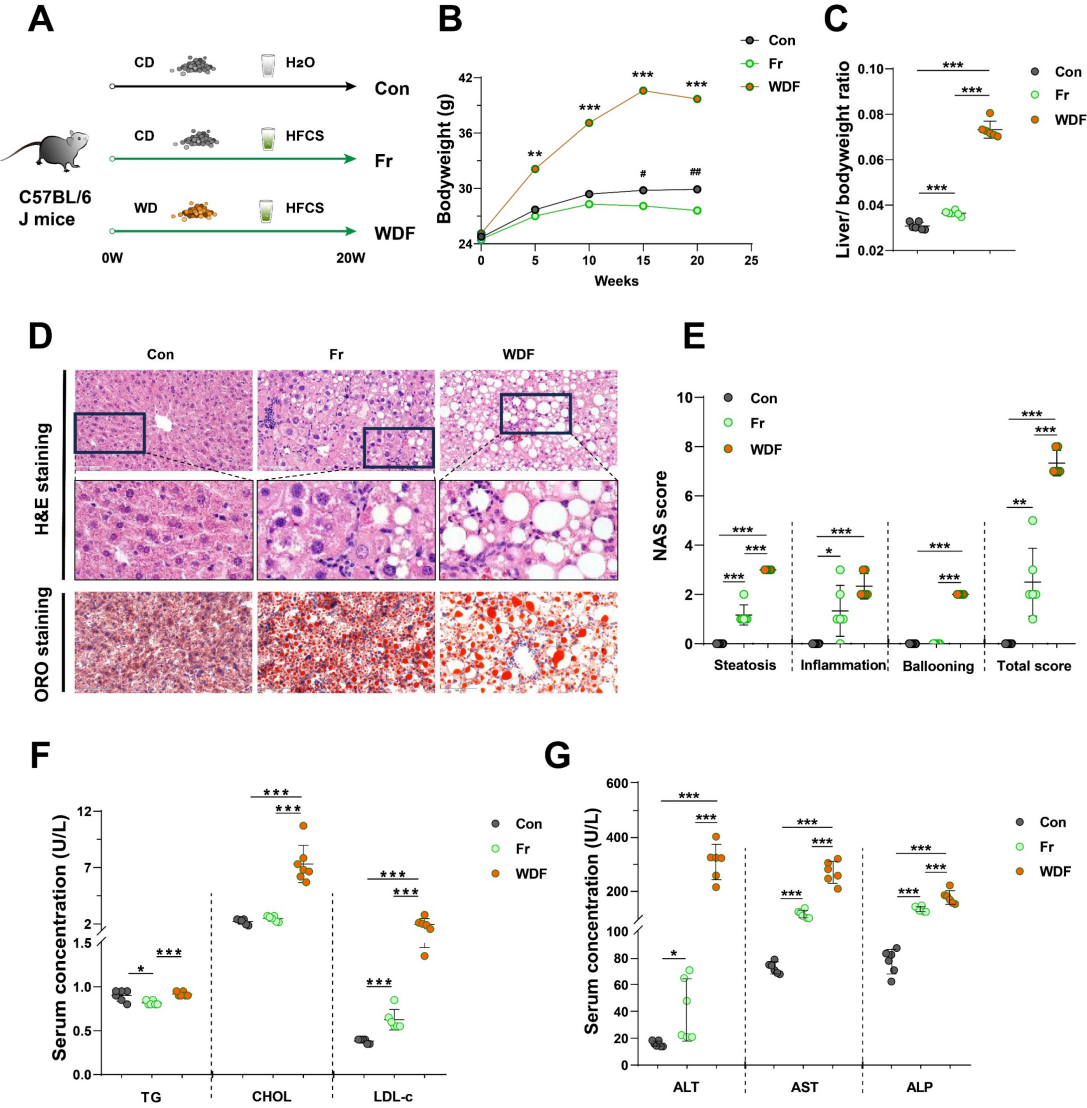
** High dietary fructose drives the development of MASLD** (A) The design of animal experiment; (B) Dynamic changes of body weight of mice; (C) Liver-to-body weight ratio; (D) Pathological staining of liver (magnification 400×): H&E staining and ORO staining of liver sections (magnification 400×); (E) NAS score; (F) Serum levels of TG, CHOL, and LDL-c; (G) Serum levels of ALT, AST, and ALP. Data are presented as mean *±* SD. Con *vs* WDF: *; Con *vs* Fr: #. ^*^p<0.05, ^**^p<0.01, ^***^p<0.001; ^#^*p*<0.05, ^##^*p*<0.01, ^###^*p*<0.001.

To investigate the impact of dietary fructose on MASLD, we fed C57BL/6J mice CD with tap water (Con group), CD with HFCS in drinking water (Fr group) or WD with HFCS in drinking water (WDF group) (**Fig. 1A**). During the 20 weeks feeding period, mice in Fr group tended to consume more water but less food (**Fig. S1A&B**), which may partially explain the body weight decrease in mice (**Fig. 1B**). However, fructose exposure resulted in increased liver-to-body weight ratio (**Fig. 1C**). High fructose administration significantly increased lipid accumulation in the liver, as evidenced by H&E and ORO staining (**Fig. 1D&E**) as well as the quantification of liver CHOL content (**Fig. S1C**). Concomitantly, serum levels of LDL-c in fructose-exposed mice were increased (**Fig. 1F**). WDF mice exhibited a more pronounced increase in body weight and hepatic lipid accumulation compared to Fr mice (**Fig. 1B-E; Fig. S1C**), indicating fructose could amplify the lipid accumulation in the liver.

High fructose consumption also resulted in increased infiltration of inflammatory cells in the liver and higher inflammatory scores (**Fig. 1D&E**). Highly activated macrophages are the typical feature of hepatic inflammation, we also observed an increase of positive staining of macrophage markers F4/80 and CD68 in the liver sections of HFCS-exposed mice (**Fig. S1D**). Furthermore, high fructose consumption promoted the secretion of inflammatory cytokines IL-1β, IL-6, and TNF-α in the liver (**Fig. S1E**). When compared with Fr mice, the WDF mice exhibited more significant hepatic inflammation and increased NAS scores (**Fig. 1D&E; Fig. S1D&E**), suggesting an additive effect between fructose and WD in promoting MASLD. Additionally, serum levels of ALT, AST, and ALP were elevated in Fr mice and further amplified when combined with WD feeding (**Fig. 1G**). We also assessed the circulating cytokines, and found increased systemic inflammatory cytokines in the circulation of fructose-exposed mice (**Fig. S1F**). Together, these results revealed that high dietary fructose could drive the development of MASLD.

### High dietary fructose promotes MASLD progression

**Figure 2:**
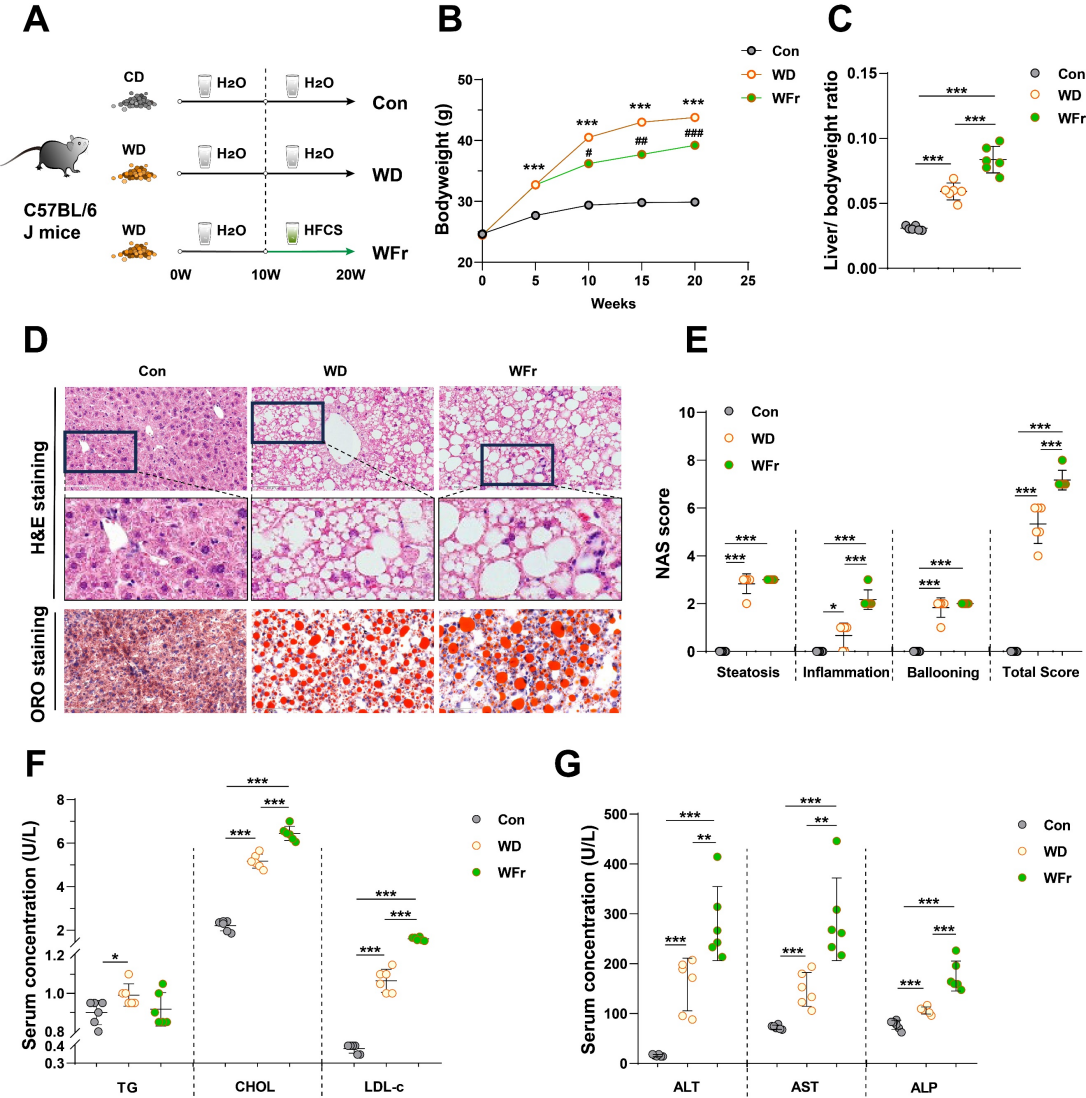
** High dietary fructose promotes the progression of MASLD** (A) Fructose induction scheme with WD feeding; (B) Dynamic changes in body weight of mice; (C) Liver-to-body weight ratio; (D) Pathological staining of liver (magnification 400×): H&E staining and ORO staining of liver sections (magnification 400×); (E) NAS score; (F) Serum levels of TG, CHOL, and LDL-c; (G) Serum levels of ALT, AST, and ALP. The quantification data are presented as mean *±* SD. Con *vs* WD: *; WD *vs* WFr: #. ^*^p<0.05, ^**^p<0.01, ^***^p<0.001; ^#^*p*<0.05, ^##^*p*<0.01, ^###^*p*<0.001.

To further elucidate the impact of high dietary fructose on MASLD, we fed the mice with 10-week WD to induce MASLD (WD group), and followed with a combination of HFCS and WD for another 10 weeks (WFr group) (**Fig. 2A**). Consistent with previous experiment, fructose-treated mice tended to consume more water and less food (**Fig. S2A&B**), and the body weight was lower than WD mice but still higher than Con mice (**Fig. 2B**). Mice in the WD group exhibited the typical MASLD phenotype (**Fig. 2C-E**), whereas WFr mice demonstrated a more pronounced increase in liver-to-body weight ratio (**Fig. 2C**). We also observed that high fructose administration resulted in aggravated lipid accumulation in the liver, as evidenced by H&E and ORO staining (**Fig. 2D&E**) and the quantification of liver CHOL content (**Fig. S2C**). Additionally, serum levels of LDL-c and CHOL were elevated in fructose-exposed mice (**Fig. 2F**). Together, these findings implied that fructose could exaggerate lipid accumulation in the liver of mice with pre-existing fatty liver.

Although the hepatic scores of steatosis and ballooning were comparable between WD and WFr mice, high fructose consumption promoted liver inflammation (**Fig. 2D&E**), infiltration of macrophages (F4/80 and CD68) (**Fig. S2D**), and production of hepatic inflammatory cytokines (**Fig. S2E**). Furthermore, serum ALT, AST, and ALP levels in WD mice were further elevated by fructose supplementation (**Fig. 2G**). WD mice showed comparable levels of systemic inflammatory cytokines (serum IL-1β, IL-6, and TNF-α) with Con mice, while these indicators were raised in WFr mice (**Fig. S2F**). Collectively, our results indicated that high dietary fructose might promote MASLD progression.

### High fructose intake upregulates hepatic USP2 expression

**Figure 3:**
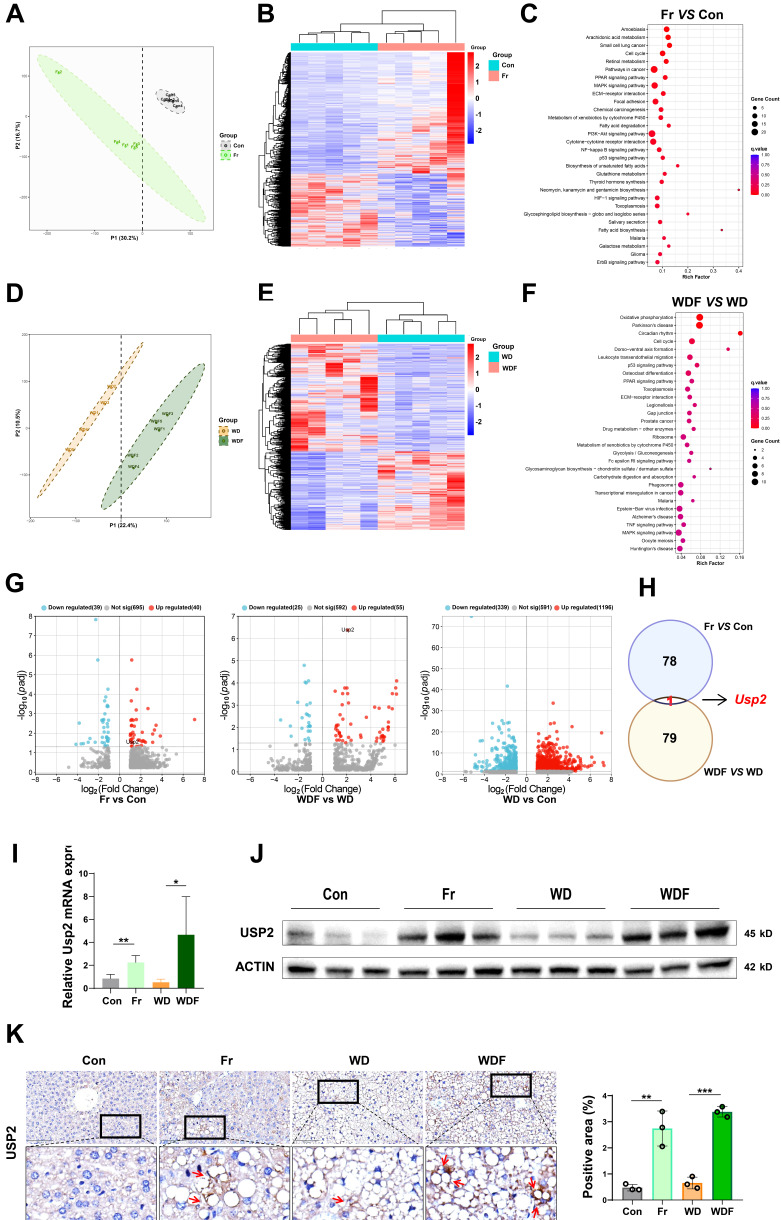
** Fructose upregulates hepatic USP2 expression** (A) Plot of PLSDA analysis between Con and Fr mice; (B) Heatmap of DEGs (*p*<0.05) between Con and Fr mice; (C) KEGG analysis between Con and Fr groups; (D) Plot of PLSDA analysis between WD and WDF groups. (E) Heatmap of DEGs (*p*<0.05) between WD and WDF groups; (F) KEGG analysis between WD and WDF groups; (G) Volcano plot of significant DEGs (*p adj*<0.05) of Fr *vs* Con, WDF* vs* WD, and WD* vs* Con, respectively; (H) Plot of co-responsive genes of Fr and WDF groups when compared to Con and WD groups, respectively; (I) Relative mRNA level of *Usp2* gene in the liver; (J) Protein blotting of USP2 in the liver; (K) The immunohistochemical staining in the liver for USP2 protein (magnification 400×) and the quantification of the positively stained area. The quantification data are presented as mean *±* SD. ^*^p<0.05, ^**^p<0.01, ^***^p<0.001.

To explore the mechanisms of how dietary fructose impacts MASLD, we performed RNA-Sequence (RNA-Seq) on the liver tissues of the mice. We firstly compared the genetic profiles of mice between the Con and Fr groups, the partial least squares discriminant analysis (PLSDA) plot showed distinguished clusters (**Fig. 3A**). Compared to the Con group, there were 816 downregulated and 1356 upregulated genes in the liver of Fr group mice (**Fig. 3B**). KEGG analysis demonstrated that these differentially expressed genes (DEGs) were enriched in pathways related to tumorigenesis, glucose and lipid metabolism, and inflammation (**Fig. 3C**). We then compared genetic profiles of mice between WD and WDF groups, distinguished clusters were observed by PLSDA plot (**Fig. 3D**). Totally, 1605 DEGs were found, of which 677 were downregulated and 928 were upregulated in WDF group (**Fig. 3E**), and they were enriched in pathways related to cellular metabolism and cycle, inflammation, and immune response (**Fig. 3F**). When we cross-check all the DEGs (*p adj*<0.05) in the 4 groups, *Usp2* was the only gene that was specifically upregulated by fructose (**Fig. 3G&H**). To verify these findings, we detected the expression of USP2 in the liver of mice. Expectably, we observed increased mRNA and protein expression of USP2 in Fr and WDF mice but not in WD mice (**Fig. 3 I&J**). Analysis of immunohistochemical images of liver sections revealed that the positive staining of USP2 was more obvious in fructose-administrated mice (**Fig. 3K**). In addition, USP2 expression was also found to be upregulated in the liver of fructose-containing (fructose-, palmitate-, and cholesterol-enriched, FPC) diet-induced MASLD mice, but not in a choline-methionine deficient diet (MCD)-induced MASLD mice (**Fig. S3A**). Together, these results suggested that USP2 might be a key mediator of fructose in driving MASLD development and progression.

### Fructose increases lipid accumulation and USP2 expression in hepatocytes

**Figure 4:**
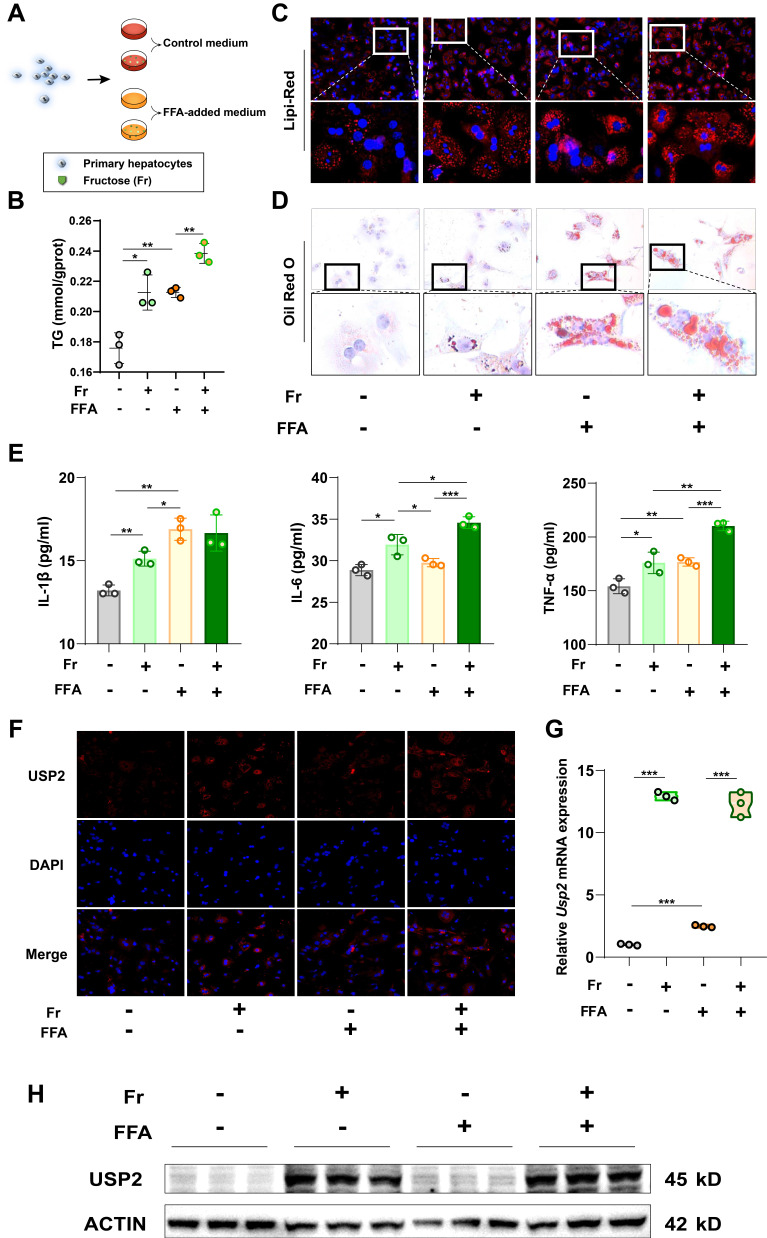
** Fructose increases lipid accumulation and USP2 expression in primary hepatocytes**. (A) The cell experiment flowchart; (B) The TG content of primary hepatocytes; (C) The Lipi-Red staining of primary hepatocytes (magnification 200×), the bottom panel figures are amplification of the upper panel; (D) The ORO staining of primary hepatocytes (magnification 200×), the bottom panel figures are amplification of the upper panel; (E) The levels of IL-1β, IL-6, and TNF-α in the culture medium of primary hepatocytes; (F) The immunofluorescence for USP2 in primary hepatocytes (magnification 100×); (G) Relative mRNA level of *Usp2* gene in primary hepatocytes; (H) Protein blotting of USP2 in primary hepatocytes. The quantification data are presented as mean *±* SD. ^*^p<0.05, ^**^p<0.01, ^***^p<0.001.

As USP2 is increased in the liver of fructose-treated mice, we then tried to determine the target cells. To investigate the basic expression of the *Usp2* gene in the liver of mice, we compared the two subtypes of primary cells (primary hepatocytes and Kupffer cells), and found that the primary hepatocytes expressed higher mRNA levels of *Usp2* in comparison to Kupffer cells (**Fig. S3B**). Since primary hepatocytes are the dominant cell type of the liver, the higher expression of *Usp2* upon fructose treatment might be the main contributor to MASLD. We cultured primary hepatocytes with fructose (Fr), free fatty acid (FFA) (oleic acid: palmitic acid =2:1), or the combination of Fr and FFA (FFr), respectively (**Fig. 4A**). We revealed that both fructose and FFA treatment increased the cellular TG levels in mouse primary hepatocytes compared with untreated Control cells (**Fig. 4B**), and enlargement of lipid droplets can be observed by Lipi-Red and ORO staining (**Fig. 4C&D**). Mouse primary hepatocytes that cultured with FFr showed more pronounced lipid accumulation than FFA-treated cells (**Fig. 4B-D**), indicating an additive effect of fructose and FFA on hepatocytes. The production of inflammatory cytokines IL-1β, IL-6, and TNF-α in the medium was also significantly increased in hepatocytes exposed to fructose, while IL-6 and TNF-α levels in FFr-treated cells were higher than in FFA cells (**Fig. 4E**). In parallel, we observed increased USP2 expression in fructose- but not in FFA-treated primary hepatocytes (**Fig. 4F-H**). Meanwhile, these results were further confirmed in murine hepatocyte cell line AML12 cells (**Fig. S4**). Collectively, these results demonstrated that fructose triggered or exacerbated lipid accumulation and USP2 expression in hepatocytes.

### Fructose induces hepatocyte steatosis and inflammation via USP2

**Figure 5:**
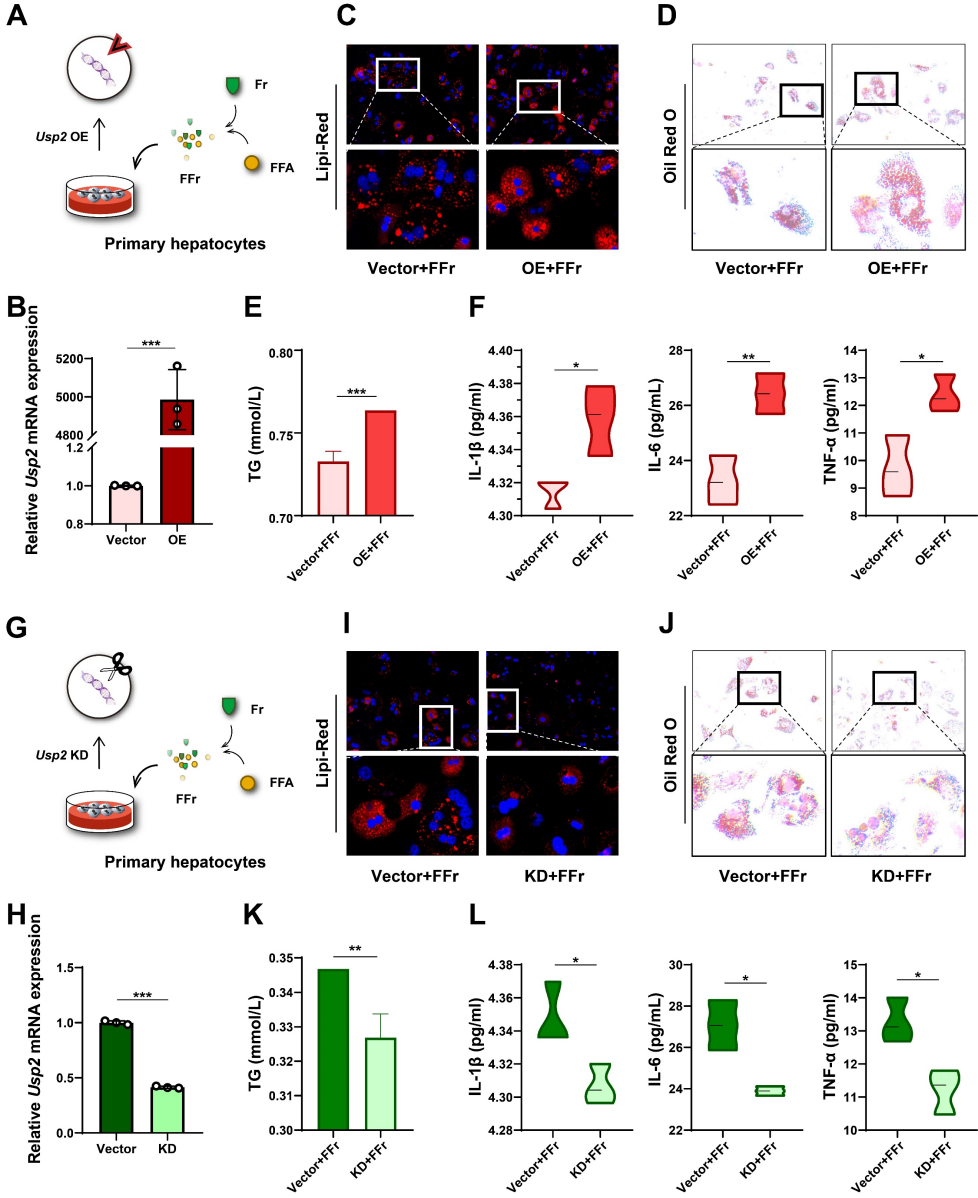
** Fructose induces hepatocyte steatosis and inflammation via USP2 in primary hepatocytes**. (A) The cell experiment flowchart of *Usp2* overexpression in primary hepatocytes; (B) The mRNA expression of *Usp2* gene in primary hepatocytes; (C) The Lipi-Red staining (magnification 200×) of *Usp2*-overexpressed primary hepatocytes, the bottom panel figures are amplification of the upper panel; (D) The ORO staining (magnification 100×) of *Usp2*-overexpressed primary hepatocytes, the bottom panel figures are amplification of the upper panel; (E) The TG content of *Usp2*-overexpressed primary hepatocytes; (F) The levels of IL-1β, IL-6, and TNF-α in the culture medium of *Usp2*-overexpressed primary hepatocytes; (G) The cell experiment flowchart of *Usp2* knockdown in primary hepatocytes; (H) The mRNA expression of *Usp2* gene in primary hepatocytes; (I) The Lipi-Red staining (magnification 200×) of *Usp2-*knockdowned primary hepatocytes, the bottom panel figures are amplification of the upper panel; (J) The ORO staining (magnification 100×) of *Usp2-*knockdowned primary hepatocytes, the bottom panel figures are amplification of the upper panel; (K) The TG content of *Usp2-*knockdowned primary hepatocytes; (L) The levels of IL-1β, IL-6, and TNF-α in the culture medium of *Usp2-*knockdowned primary hepatocytes. The quantification data are presented as mean *±* SD. ^*^p<0.05, ^**^p<0.01, ^***^p<0.001.

To confirm the role of USP2 in fructose-induced steatosis and inflammation, we overexpressed the *Usp2* gene in primary hepatocytes, and subsequently treated these cells with FFr (**Fig. 5A**). The upregulation of *Usp2* expression confirmed the success of gene transfection (**Fig. 5B**). *Usp2* overexpressed primary hepatocytes showed comparable cellular TG content but increased IL-1β levels in comparison with vector-transfected cells when cultured in the conventional medium (**Fig S5A&B**). Upon FFr treatment, the number and the size of lipid droplets were further increased (**Fig. 5C&D**), and cellular TG content was significantly augmented (**Fig. 5E**) in *Usp2*-overexpressed cells. Meanwhile, *Usp2*-overexpressed hepatocytes displayed aggressive inflammation, as evidenced by increased production of inflammatory cytokines IL-1β, IL-6, and TNF-α (**Fig. 5F**). On the contrary, *Usp2* knockdown (**Fig. 5G&H**) resulted in decreased size and number of lipid droplets (**Fig. 5I&J**), and reduced TG content (**Fig. 5K**) in FFr-exposed primary hepatocytes. Concurrently, *Usp2* deficiency also decreased the secretion of inflammatory cytokines, including IL-1β, IL-6, and TNF-α, in mouse primary hepatocytes (**Fig. 5L**). While *Usp2* knockdown showed comparable TG content but decreased the IL-1β levels with vector-transfected primary hepatocytes when cultured in the conventional medium (**Fig.S5C&D**). These findings were also confirmed by the results in AML12 cells (**Fig. S5&6**). Overall, the above results suggested that fructose induces hepatocyte steatosis and inflammation via USP2.

### The function of USP2 is dependent on C/EBPα/ 11β-HSD1

**Figure 6:**
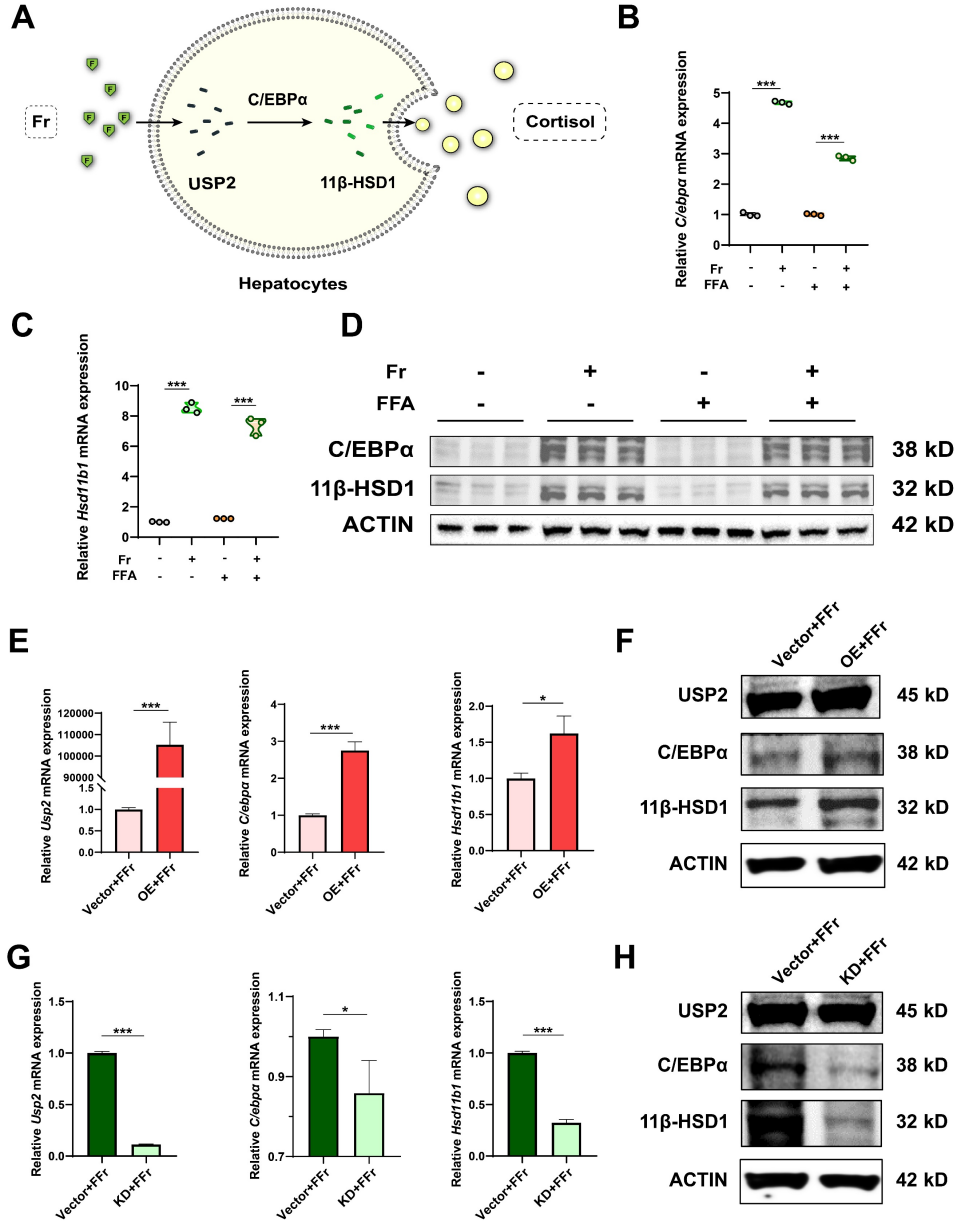
** The function of USP2 depends on C/EBPα/ 11β-HSD1 in fructose-stressed primary hepatocytes**. (A) Hypothesis diagram for USP2/ 11β-HSD1 pathway in the liver; (B, C) Relative mRNA expression of C/EBPα (*C/ebpα*) and 11β-HSD1 (*Hsd11b1*) in hepatocytes; (D) Protein blotting of C/EBPα and 11β-HSD1 in hepatocytes; (E) Relative mRNA expression of USP2 (*Usp2*), C/EBPα (*C/ebpα*), and 11β-HSD1 (*Hsd11b1*) in *Usp2*-overexpressed hepatocytes; (F) Protein blotting of USP2, C/EBPα, and 11β-HSD1 in *Usp2*-overexpressed hepatocytes; (G) Relative mRNA expression of USP2 (*Usp2*), C/EBPα (*C/ebpα*), and 11β-HSD1 (*Hsd11b1*) in *Usp2*-knockdowded hepatocytes; (H) Protein blotting of USP2, C/EBPα, and 11β-HSD1 in *Usp2*-knockdowded hepatocytes. The quantification data are presented as mean *±* SD. ^*^p<0.05, ^**^p<0.01, ^***^p<0.001.

Previous research indicates that USP2 stabilizes C/EBPα, potentially leading to the upregulation of 11β-HSD1 expression in hepatocytes, which catalyzes the regeneration of active glucocorticoids^16^, ^17^, ^28^-^31^. C/EBPα is a potent transcriptional factor of 11β-HSD1 in the liver, and activation of the USP2/ C/EBPα/ 11β-HSD1 signaling may account for fructose effects on MASLD (**Fig. 6A**). We detected the potential downstream molecules of USP2 in primary hepatocytes treated with fructose, the expression of C/EBPα (*C/ebpα*) and 11β-HSD1 (*Hsd11b1*) was significantly increased both at mRNA (**Fig. 6B&C**) and protein level (**Fig. 6D**). However, the expression of C/EBPα and 11β-HSD1 was not statistically different between FFA-treated cells and control cells (**Fig. 6B-D**), suggesting the activation of C/EBPα and 11β-HSD1 signaling specifically occurred in fructose-treated cells. Additionally, we observed the same trend of changes in AML12 cells (**Fig. S7A-C**). Hepatocytes with *Usp2* overexpression showed aggressive lipid accumulation and secretion of inflammatory cytokines (**Fig. 5C-F**), and the expression of C/EBPα (*C/ebpα*) and 11β-HSD1 (*Hsd11b1*) was also increased (**Fig. 6E&F**) upon fructose challenge. In contrast, *Usp2-*knockdown induced improvement of steatosis and inflammation (**Fig. 5I-L**), and downregulation of C/EBPα (*C/ebpα*) and 11β-HSD1 (*Hsd11b1*) expression in these cells (**Fig. 6G&H**). Consistent results were obtained in FFr-challenged AML12 cells with* Usp2* overexpression or knockdown (**Fig. S7D-G**). Hence, we concluded that USP2 regulates steatosis and inflammation depending on the C/EBPα/ 11β-HSD1 pathway*.*

### C/EBPα/ 11β-HSD1 signaling is activated in high fructose-induced MASLD mice

**Figure 7:**
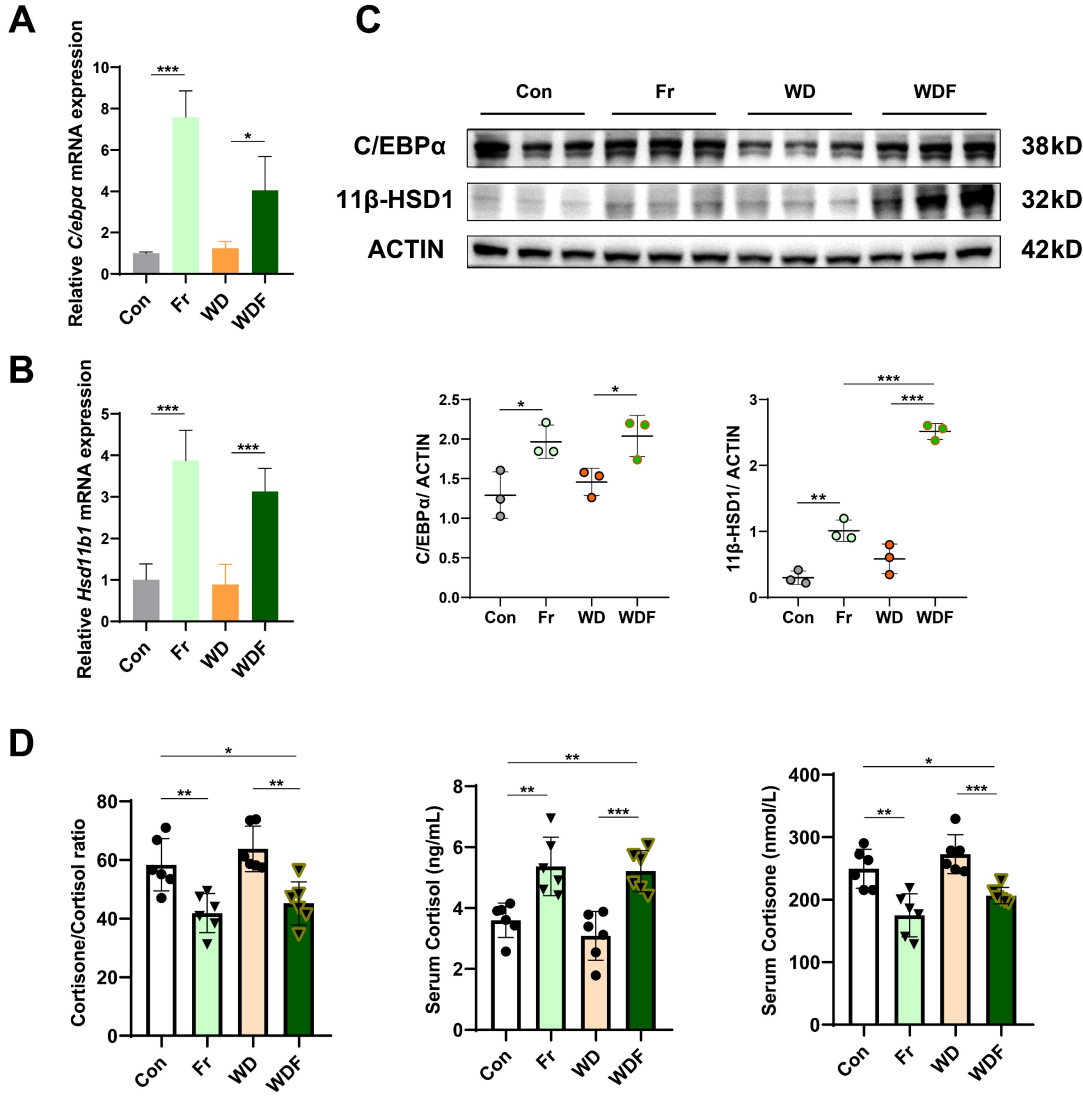
** Fructose induces MASLD via the USP2/ 11β-HSD1 pathway** (A, B) Relative mRNA expression of C/EBPα (*C/ebpα*) and 11β-HSD1 (*Hsd11b1*) in the liver of the mice; (C) Protein blotting and statistical analysis of C/EBPα and 11β-HSD1 in the liver of the mice; (D) Serum levels of cortisol and cortisone, and cortisone-to-cortisol ratio. The quantification data are presented as mean *±* SD. ^*^p<0.05, ^**^p<0.01, ^***^p<0.001.

To validate that USP2 mediated the C/EBPα/ 11β-HSD1 pathway, we detected their expression in the liver of HFCS-drinking mice. Consistent with *in vitro* findings, the expression of C/EBPα (*C/ebpα*) and 11β-HSD1 (*Hsd11b1*) were significantly increased in Fr and WDF groups (**Fig. 7A-C**). 11β-HSD1 is a widely distributed reductase, it regulates energy metabolism and inflammation by converting inactive cortisone into active cortisol, and the liver is the most important source of visceral cortisol^32^-^34^. Therefore, we assessed the level of hormones, and found that fructose administration significantly increased serum cortisol levels and reduced cortisone levels (**Fig. 7D**). Correspondingly, the cortisol/ cortisone ratio was decreased, whereas the cortisol and cortisone levels were comparable between Con and WD mice (**Fig. 7D**). To further confirm the impact of cortisol on MASLD, we treated hepatocytes with cortisol, and found that cortisol exaggerated lipid accumulation in FFA- and Fr treated primary hepatocytes and AML12 cells (**Fig. S8**). Collectively, the above results suggested that high fructose might promote MASLD formation and progression via USP2/ C/EBPα/ 11β-HSD1 signaling pathway in the liver.

## Discussion

In the present study, we found that high fructose promotes MASLD development and progression in C57BL/5J mice, and identified that USP2-mediated 11β-HSD1 signaling in the liver is crucial for MASLD induced by high fructose exposure.

High fructose consumption is widely recognized as a contributor to metabolic disorders. A cross-sectional survey among 283 Lebanese adults pinpointed an average fructose consumption of about 52g/day, comprising approximately one-tenth of total caloric intake^35^. Human physiology, however, confronts challenges when metabolizing fructose beyond 25g/day, with industrially processed fructose, noted for its concentration and swift absorption, being particularly problematic^36^. Research indicates that absorbed fructose can catalyze hepatic DNL both directly and indirectly, whereas unabsorbed fructose threatens metabolic homeostasis by forming glycation end products with proteins in the intestine 37, 38. Subsequently, attempts to curtail fructose absorption and its metabolic effects have produced varied results in preventing or managing MASLD^39^-^42^.

Natural fructose obtained from plants typically confers metabolic benefits due to its slower absorption rate and the presence of beneficial plant fiber and antioxidants. In contrast, industrial fructose sources such as HFCS and sucrose, particularly in liquid form, are rapidly absorbed and implicated in hepatic IR and MASLD^43^-^45^. Notably, HFCS-rich drinks nearly triple the likelihood of developing MASLD^7^. Animal models demonstrate that HFCS exposure heightens body adiposity, hepatic TG, and expression of DNL genes^46^. Moreover, high-fructose feeding induces glucose intolerance, IR, steatosis, hypoadiponectinemia, and mitochondrial disruption in mice^46^, ^47^. At the cellular level, HFCS additionally raises lipid deposition, oxidative stress, endoplasmic reticulum stress, and disrupts intracellular calcium^47^. Specifically, fructose triggers endoplasmic reticulum stress via the PIDDosome-sterol regulatory element binding protein (SREBP) cleavage activating protein (SCAP) axis in hepatocytes, potentially facilitating the shift from simple steatosis to steatohepatitis^48^. Moreover, fructose stimulates the secretion of inflammatory cytokines by disrupting cellular metabolic adaptation and activates the reactive oxygen species (ROS)/ NF-κB signaling pathway in macrophages^49^,^50^. Our observations in this study further expanded the previous findings, and demonstrated that high fructose intake not only instigates MASLD but also aggravates its progression in murine models.

By scrutinizing the RNA-Seq data, we unveiled a plethora of fructose-responsive genes, potentially influencing energy metabolism, inflammation, and even carcinogenesis. A noteworthy finding was the consistent upregulation of *Usp2* gene in the fructose-treated mice, irrespective of whether a CD or a WD feeding. USP2, a deubiquitinating enzyme widely expressed by mammalian cells, is implicated in various cellular processes^51^, ^52^. Studies indicate that USP2 fosters tumor growth and spread through regulating apoptosis, autophagy, and oncoprotein stabilization^53^-^56^. Moreover, USP2 upregulation has been associated with increased liver gluconeogenesis and glucose intolerance, enhanced lipogenesis, and amplified inflammatory responses^16^-^18^, ^57^. Consistent with our *in vivo* observations, USP2 was found to be upregulated in fructose-exposed hepatocytes *in vitro*. Additionally, hepatocytes overexpressing *Usp2* exhibited increased lipid accumulation and elevated secretion of inflammatory cytokines. Conversely, *Usp2* depletion caused diminished steatosis and inflammation. These findings lead to the compelling conclusion that USP2 occupies a pivotal position in fructose-induced liver damage.

USP2 is also recognized for its role in amplifying 11β-HSD1 through the action on its substrate, C/EBPα^17^, ^28^. Research indicates that liver-specific deletion of 11β-HSD1 abrogates the gluconeogenic effects of USP2^16^. 11β-HSD1, predominantly present in the liver, is crucial for converting inactive glucocorticoids into their bioactive forms, thus playing a critical role in maintaining glucocorticoid homeostasis 58. Deubiquitination of C/EBPα by USP2 does not simply affect the stability of C/EBPα, since ubiquitination of transcription factors also acts on transcriptional functions^59^. Corresponding with these findings, fructose exposure was seen to boost C/EBPα mRNA levels in hepatocytes. Accumulating evidence implies that 11β-HSD1 activation could contribute to hepatic steatosis, inflammation, and fibrosis^29^-^31^. Decreased adiponectin levels, IR, hypercortisolism, and systemic inflammation are also linked to excessive 11β-HSD1 activity^60^, ^61^. Clinical trials have demonstrated the safety and efficacy of 11β-HSD1 inhibition in ameliorating liver steatosis and IR^29^, ^62^, ^63^. Furthermore, 11β-HSD1 inhibition mitigates hyperlipidemia, liver inflammation, and fibrosis in MASLD models^30^, ^64^-^67^. And *Hsd11b1* (11β-HSD1) gene deficiency shields mice against glucose intolerance, dyslipidemia, and obesity^68^. Our research indicates that fructose exposure leads to the upregulation of C/EBPα and 11β-HSD1, unveiling a mechanism by which USP2-mediated activation of C/EBPα and 11β-HSD1 is implicated in the onset and progression of MASLD.

The liver is the central viscus for converting inactive cortisone into active cortisol via 11β-HSD1^32^-^34^. Cortisol, the principal glucocorticoid, is widely recognized to cause various metabolic concerns. Elevated cortisol levels are closely associated with central obesity, IR, diabetes mellitus, dyslipidemia, hypertension, and subsequently a heightened risk of cardiovascular complications^69^-^71^. Notably, MASLD patients exhibit notably higher concentrations of cortisol, implicating the accentuated cortisol synthesis mediated by 11β-HSD1 contributing to MASLD^72^, ^73^. In our investigations, fructose administration led to a substantial rise in circulating cortisol and a concomitant reduction in cortisone levels. Consequently, the cortisone-to-cortisol ratio is decreased in fructose-exposed mice, indicating heightened activation of 11β-HSD1 in this context. These observations underscore the notion that disruption in glucocorticoid metabolism by 11β-HSD1 is implicated in MASLD, especially in the context of excessive fructose consumption.

While our exploration elucidated significant aspects of fructose-induced MASLD and the USP2/ 11β-HSD1 signaling pathway, it is crucial to recognize certain limitations for a thorough comprehension of these findings. Firstly, our study focuses on hepatocytes; nonetheless, it is important to consider the potential role of macrophage activation in the development of fructose-induced MASLD. While hepatocytes do dominate liver cell populations and exhibit a higher USP2 expression which bolsters our results, further research is imperative to ascertain the contributions of other liver cell types to MASLD pathology. Secondary to this, the intestine is the initial site of fructose uptake and metabolism^38^. Therefore, the effects of fructose on the intestinal milieu and its microbiota are important aspects warranting further exploration. Future research endeavors should aim to decipher the complex interactions between dietary fructose, intestinal health, and the gut microbiome. Thirdly, our study discusses the well-documented correlation between 11β-HSD1 activity and cortisol conversion - a notion that is broadly acknowledged within the field 74. Nevertheless, the validity of this association would be significantly reinforced by targeted pharmacological or genetic studies. Lastly, considering the widespread expression of USP2, the prospective impact of cross-talk between organs on MASLD onset and progression warrants further scrutiny. The systemic implications of these inter-organ interactions present another layer of complexity to MASLD etiology and pathology, which should be the focus of future research endeavors.

In summary, our findings indicate that high fructose consumption drives and exaggerates MASLD, pointing to the USP2/ 11β-HSD1 pathway as a key player. These insights into the relationship between diet and liver health highlight the multifaceted nature of MASLD's etiology. Notably, our findings identified USP2 as a pivotal molecular target, presenting novel therapeutic opportunities for MASLD intervention. Certainly, these findings have the potential to deepen our understanding of metabolic liver diseases and bolster progress in clinical management and drug development.

## Supplementary Material

Supplementary figures.

## Figures and Tables

**Table 1:** Plasma information

P-Code	Product
138571-40541 GM-C34480	NC AML12 Cell Line (6716)
138571-40541 GM-C34481	Mouse-USP2 AML12 Cell Line (79157)
138571-40541 GM-C34727	NC AML12 Cell Line (2469)
138571-40541 150827	Mouse-USP2-ShRNA1545 AML12 Cell Line (79156)

**Table 2:** Primer sequence for RT-qPCR

Gene	F	R	bp
*Usp2*	CGATTGTGGCTACTGCTCTACAG	CAGCAAGTTGGCTTCTCATCAC	153
*C/ebpa*	CAAGAACAGCAACGAGTACCG	GTCACTGGTCAACTCCAGCAC	124
*Hsd11b1*	GCCTTGAACTCGGAGCAGC	TTCGCACAGAGTGGATGTCG	180
*β-actin*	TGACGTGGACATCCGCAAAG	CTGGAAGGTGGACAGCGAGG	205

## Abbreviations

ALP: alkaline phosphatase
ALT: alanine aminotransferase
AST: aspartate aminotransferase
CD: chow diet
C/EBPα: CCAAT/enhancer binding protein alpha
CHOL: total cholesterol
DEGs: differentially expressed genes
DNL: *de novo* lipogenesis
FFA: free fatty acid
FFr: the combination of Fr and FFA
Fr: fructose
HCC: hepatocellular carcinoma
H&E staining: hematoxylin and eosin staining
HFCS: high fructose corn syrup
HFD: high-fat diet
IDOL: inducible degrader of the LDLR
IHC: immunohistochemical
IL: interleukin
IR: insulin resistance
KD: knockdown
LDL-c: low density lipoprotein-cholesterol
LDLR: low-density lipoprotein receptor
MAFLD: metabolic dysfunction-associated fatty liver disease
MASLD: metabolic dysfunction-associated steatotic liver disease
MASH: metabolic dysfunction-associated steatohepatitis
NAFLD: non-alcoholic fatty liver disease
NF-κB: nuclear factor kappaB
OE: overexpression
ORO staining: oil red O staining
PLSDA: partial least squares discriminant analysis
RT-qPCR: real-time quantitative PCR
RNA-Seq: RNA-sequencing
SCAP: sterol regulatory element binding protein (SREBP) cleavage activating protein
TG: triglycerides
TNF-α: tumor necrosis factor alpha: UA: uric acid
USP2: ubiquitin-specific peptidase 2
WD: western diet
11β-HSD1: 11beta-hydroxysteroid dehydrogenase type 1
