# Supplementary material for: High dietary Fructose Drives Metabolic Dysfunction-Associated Steatotic Liver Disease via Activating ubiquitin-specific peptidase 2/11β-hydroxysteroid dehydrogenase type 1 Pathway in Mice
Source: Int J Biol Sci. 2024 Jun 17;20(9):3480–96. doi: 10.7150/ijbs.97309 (PMC11234208; doi:10.7150/ijbs.97309)
Supplement: Supplementary file 1 — Supplementary figures. [file ijbsv20p3480s1.pdf]

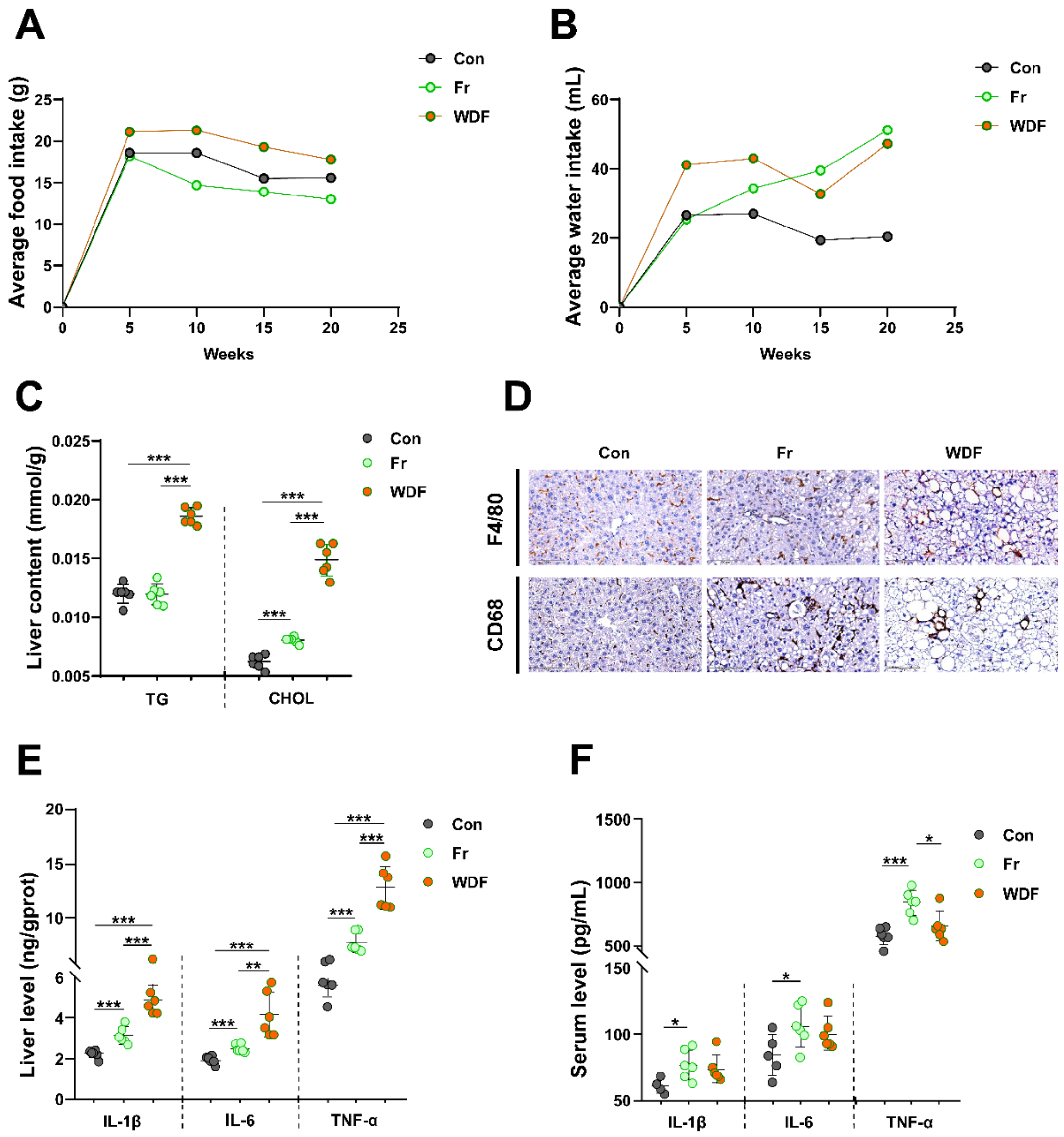

Figure S1. **High dietary fructose drives the development of MASLD**

(A-B) Dynamic changes in average water intake and average food consumption in mice; (C) Hepatic lipid content; (D) The immunohistochemical staining of liver (magnification 400×) for F4/80 and CD68; (E) Levels of hepatic IL-1β, IL-6, and TNF-α; (F) Serum levels of IL-1β, IL-6, and TNF-α. The quantification data are presented as mean ± SD. $^{*}p<0.05$, $^{**}p<0.01$, $^{***}p<0.001$

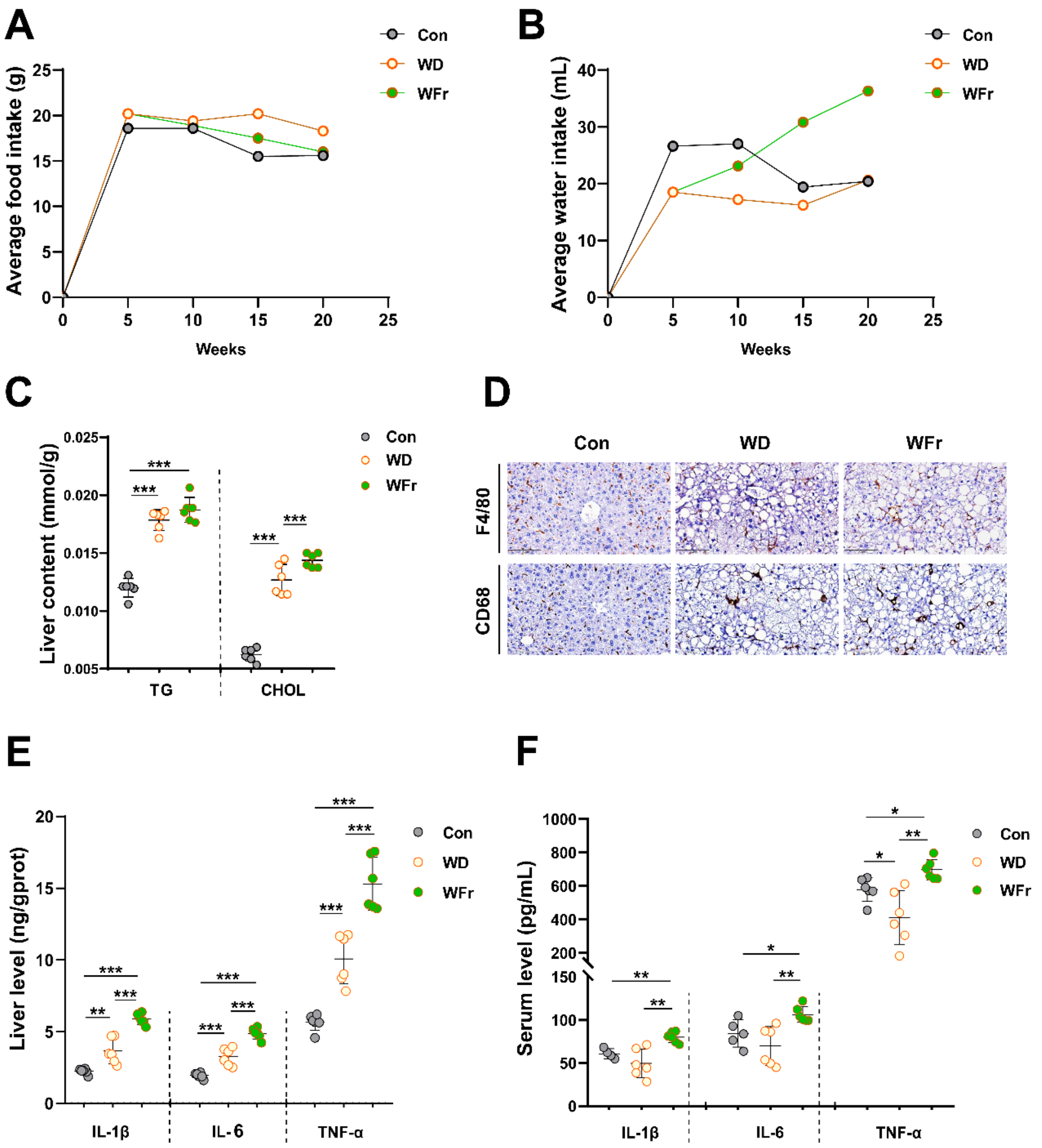

Figure S2. High dietary fructose promotes the progression of MASLD

(A-B) Dynamic changes in average water intake and average food consumption in mice; (C) Hepatic lipid content; (D) The immunohistochemical staining of the liver tissue (magnification 400×) for F4/80 and CD68; (E) Levels of hepatic IL-1β, IL-6, and TNF-α; (F) Serum levels of IL-1β, IL-6, and TNF-α. The quantification data are presented as mean ± SD. $^{*}p<0.05$, $^{**}p<0.01$, $^{***}p<0.001$

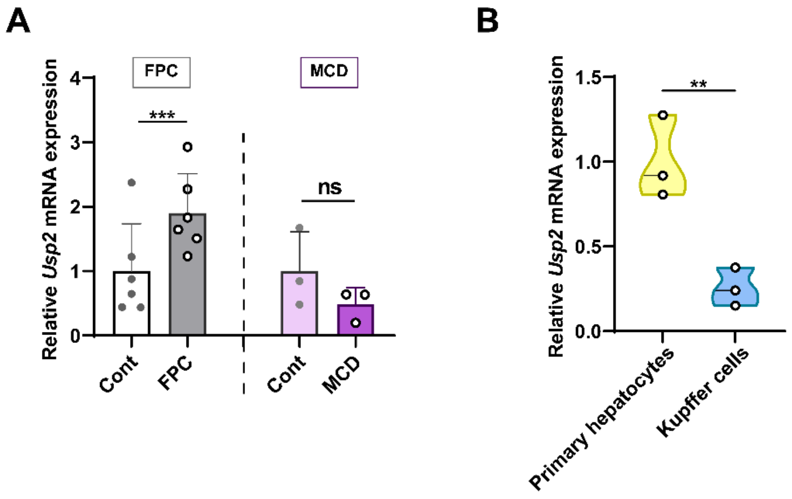

Figure S3 Expression of USP2 in different animal models and liver cells

(A) Relative mRNA level of *Usp2* in the liver between FPC and MCD model mice; (B) Relative mRNA level of *Usp2* in primary hepatocytes and Kupffer cells isolated from wild-type mice. The quantification data are presented as mean ± SD. $^{**}p<0.01$, $^{***}p<0.001$

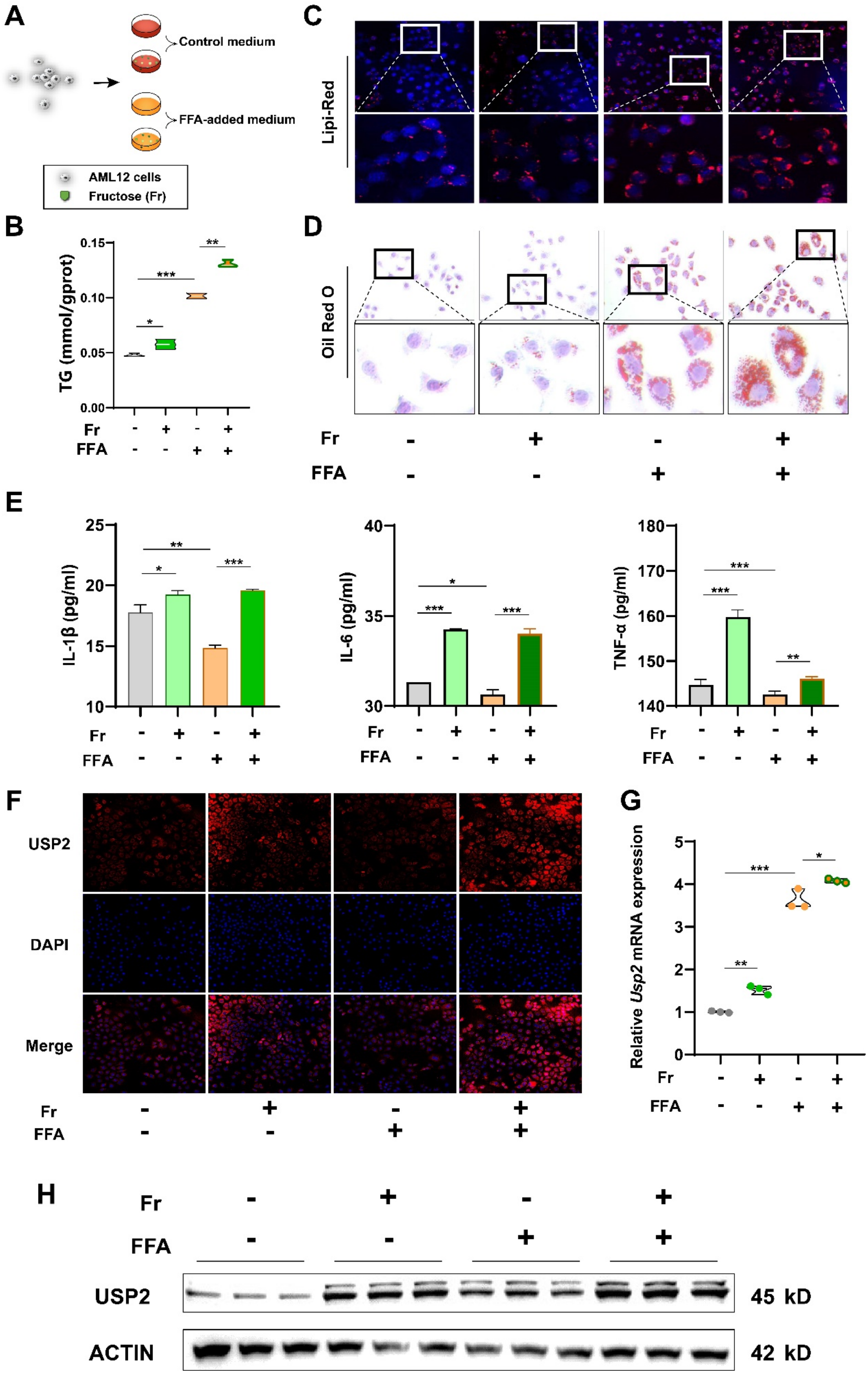

Figure S4. Fructose increases lipid accumulation and USP2 expression in AML12 cells

(A) The cell experiment flowchart; (B) The TG content of AML12 cells; (C) The Lipi-Red staining of AML12 cells (magnification 200×), the bottom panel figures are amplification of the upper panel figures; (D) The ORO staining of AML12 cells (magnification 200×), the bottom panel figures are amplification of the upper panel figures; (E) The levels of IL-1β, IL-6, and TNF-α in the culture medium of AML12 cells; (F) The immunofluorescence for USP2 in AML12 cells (magnification 100×); (G) Relative mRNA level of *Usp2* gene in AML12 cells; (H) Protein blotting of USP2 in AML12 cells. The quantification data are presented as mean ± SD. $^{*}p<0.05$, $^{**}p<0.01$, $^{***}p<0.001$

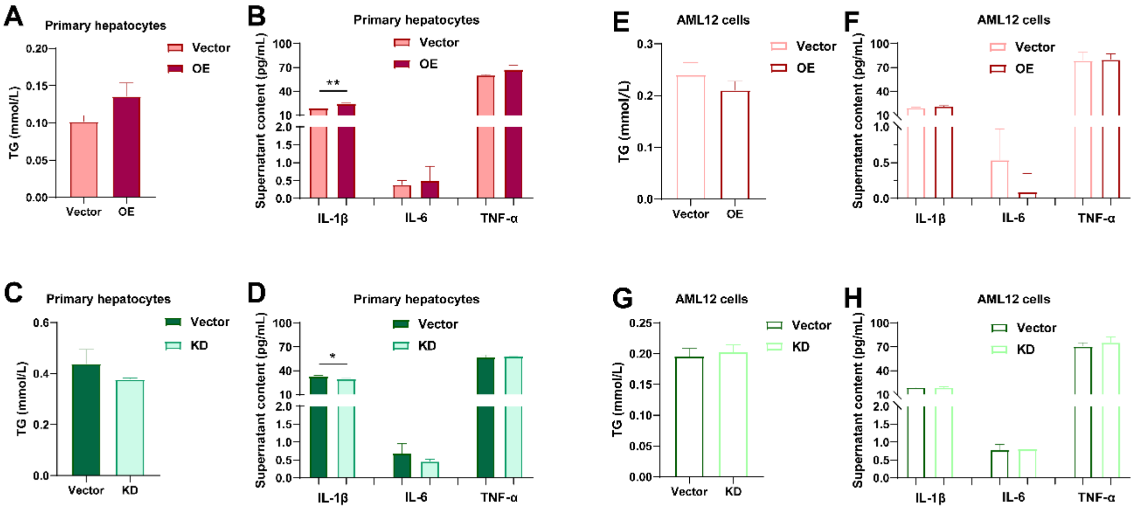

Figure S5. The effects of USP2 overexpression and USP2 knockdown on untreated hepatocytes

(A) The TG content of *Usp2*-overexpressed primary hepatocytes; (B) The levels of IL-1β, IL-6, and TNF-α in the medium of *Usp2*-overexpressed primary hepatocytes; (C) The TG content of primary hepatocytes with *Usp2* knockdown; (D) The levels of IL-1β, IL-6, and TNF-α in the medium of primary hepatocytes with *Usp2* knockdown; (E) The TG content of *Usp2*-overexpressed AML12 cells; (F) The levels of IL-1β, IL-6, and TNF-α in the medium of *Usp2*-overexpressed AML12 cells; (G) The TG content of AML12 cells with *Usp2* knockdown; (H) The levels of IL-1β, IL-6, and TNF-α in the medium of AML12 cells with *Usp2* knockdown. The quantification data are presented as mean ± SD. $^{*}p<0.05$, $^{**}p<0.01$

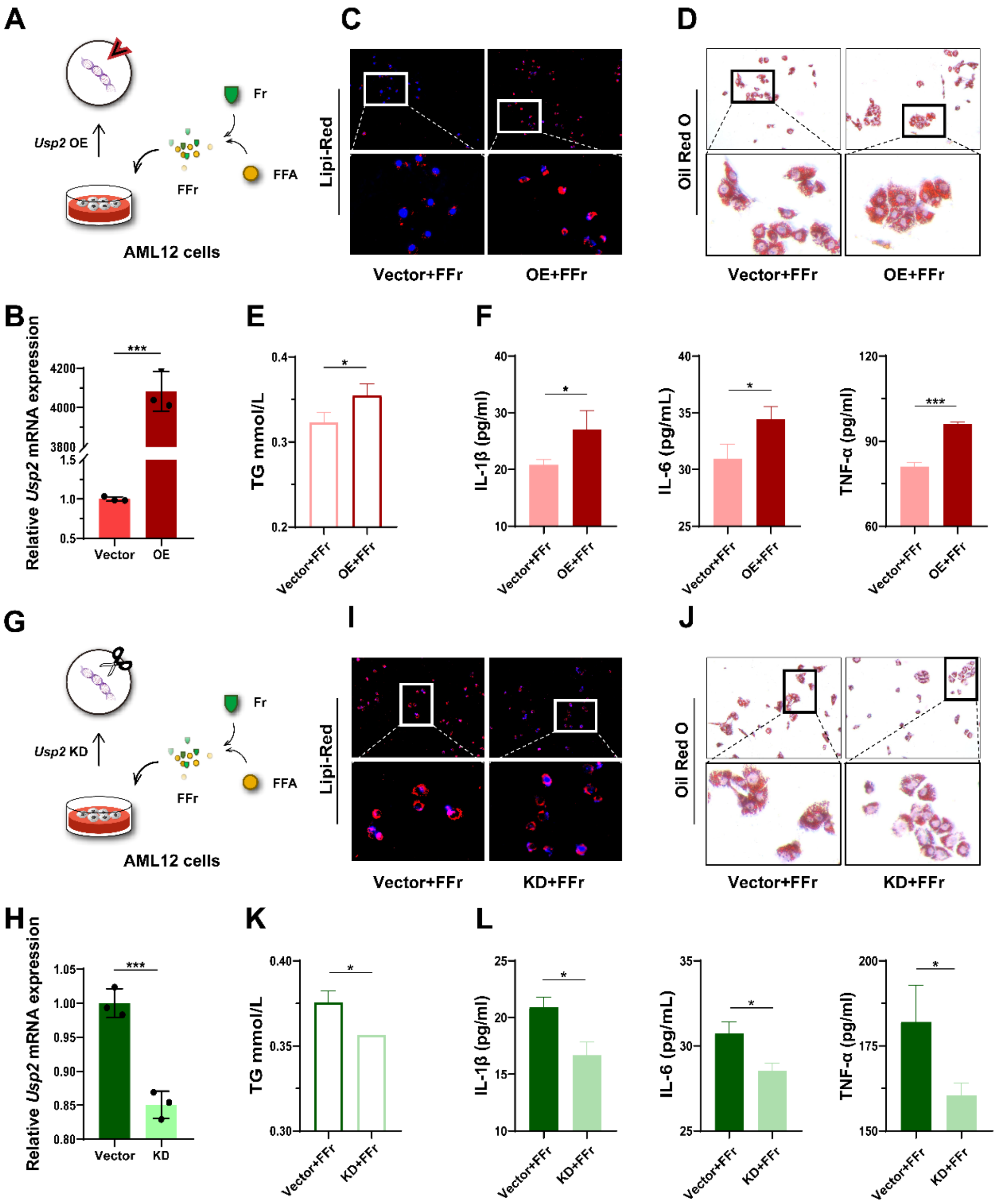

Figure S6. Fructose induces steatosis and inflammation via USP2 in AML12 cells

(A) The cell experiment flowchart of *Usp2* overexpression in AML12 cells; (B) The mRNA expression of *Usp2* gene in AML12 cells; (C) The Lipi-Red staining (magnification 200×) of *Usp2*-overexpressed AML12 cells, the bottom panel figures are amplification of the upper panel figures; (D) The ORO staining (magnification 100×) of *Usp2*-overexpressed AML12 cells, the bottom panel figures are amplification of the upper panel figures; (E) The TG content of *Usp2*-overexpressed AML12 cells; (F) The levels of IL-1β, IL-6, and TNF-α in the culture medium of *Usp2*-overexpressed AML12 cells; (G) The cell experiment flowchart of *Usp2* knockdown in AML12 cells; (H) The mRNA expression of *Usp2* gene in AML12 cells; (I) The Lipi-Red staining (magnification 200×) of *Usp2*-knockdowned AML12 cells, the bottom panel figures are amplification of the upper panel figures; (J) The ORO staining (magnification 100×) of *Usp2*-

knockdowned AML12 cells, the bottom panel figures are amplification of the upper panel figures; (K) The TG content of *Usp2*-knockdowned AML12 cells; (L) The levels of IL-1β, IL-6, and TNF-α in the culture medium of *Usp2*-knockdowned AML12 cells. The quantification data are presented as mean ± SD. $^{*}p < 0.05$, $^{**}p < 0.01$, $^{***}p < 0.001$

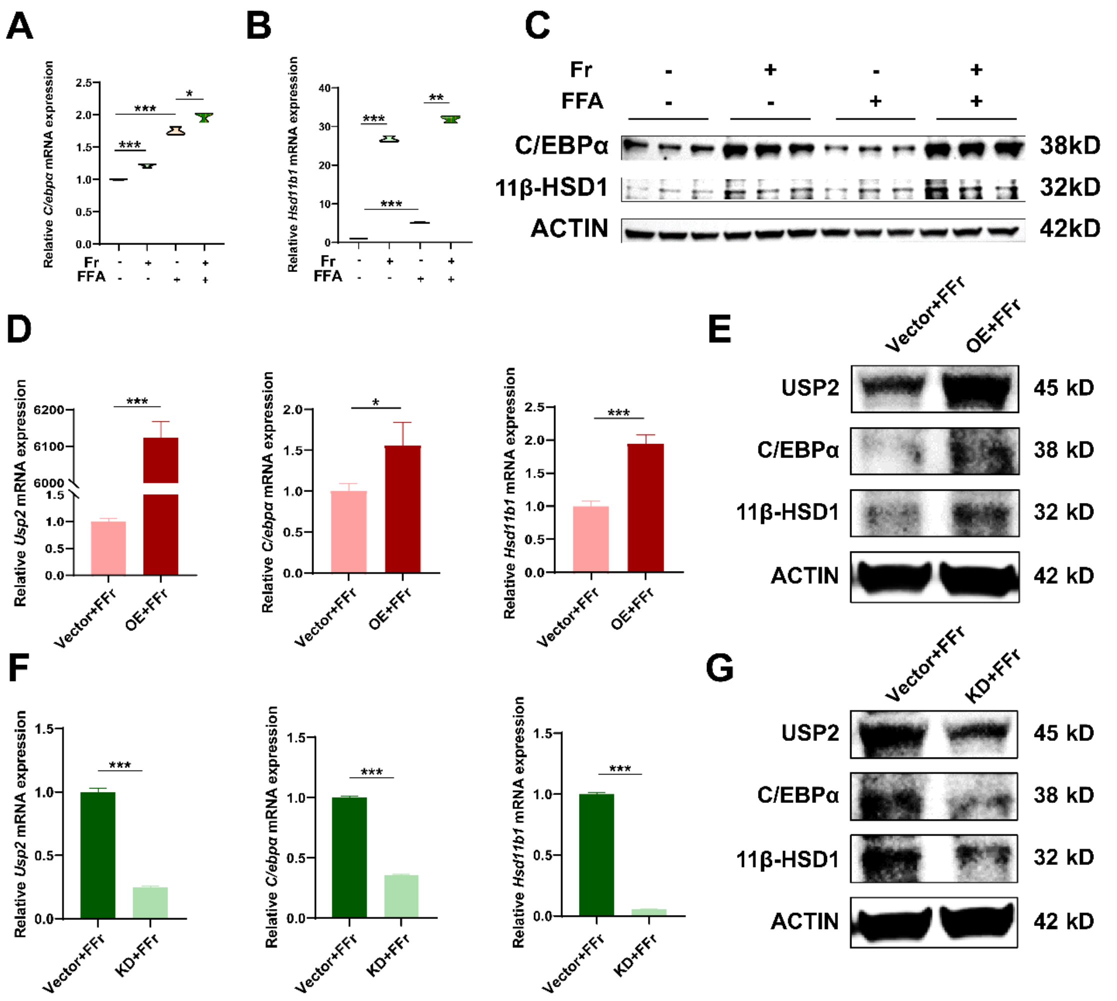

Figure S7. The function of USP2 depends on C/EBPα/ 11β-HSD1 in fructose-stressed AML12 cells

(A, B) Relative mRNA expression of C/EBPα (*C/ebpα*) and 11β-HSD1 (*Hsd11b1*) in AML12 cells; (C) Protein blotting of C/EBPα and 11β-HSD1 in AML12 cells; (D) Relative mRNA expression of USP2 (*Usp2*), C/EBPα (*C/ebpα*), and 11β-HSD1 (*Hsd11b1*) in *Usp2*-overexpressed AML12 cells; (E) Protein blotting of USP2, C/EBPα, and 11β-HSD1 in *Usp2*-overexpressed AML12 cells; (F) Relative mRNA expression of USP2 (*Usp2*), C/EBPα (*C/ebpα*), and 11β-HSD1 (*Hsd11b1*) in *Usp2*-knockdowded AML12 cells; (G) Protein blotting of USP2, C/EBPα, and 11β-HSD1 in *Usp2*-knockdowded AML12 cells. The quantification data are presented as mean ± SD. $^{*}p<0.05$, $^{**}p<0.01$, $^{***}p<0.001$

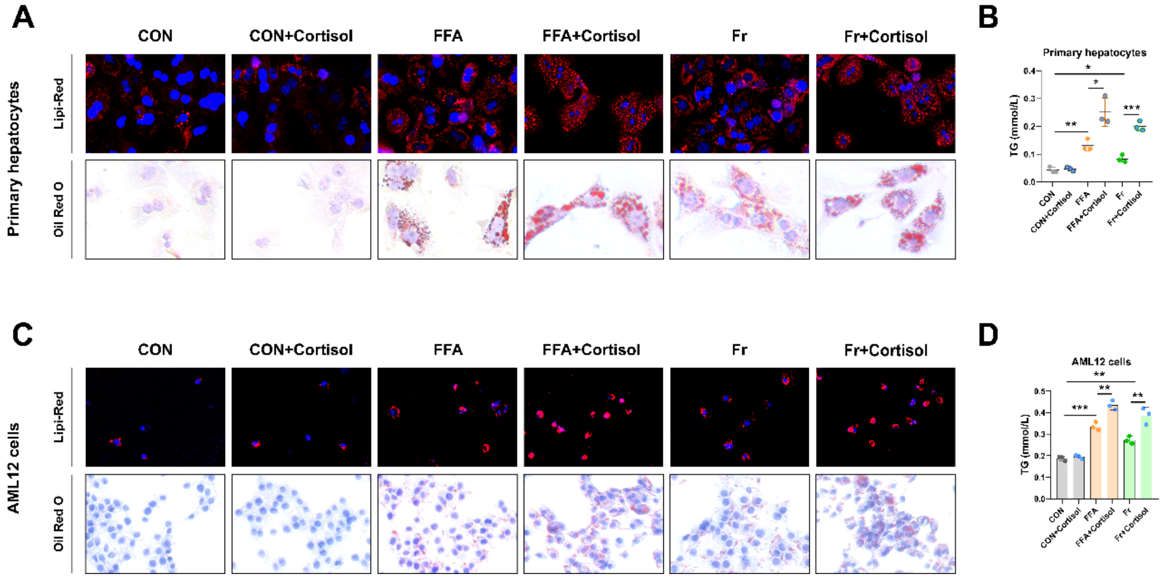

Figure S8. The effects of cortisol on lipid accumulation in hepatocytes

(A) The Lipi-Red (magnification 200×) and ORO staining (magnification 200×) of primary hepatocyte; (B) The TG content of primary hepatocytes; (C) The Lipi-Red (magnification 100×) and ORO staining (magnification 200×) of AML12 cells; (D) The TG content of AML12 cells. The quantification data are presented as mean ± SD. $^{*}p<0.05$, $^{**}p<0.01$, $^{***}p<0.001$
